# Counseling practice of community pharmacists for diabetes mellitus patients in Addis Ababa, Ethiopia

**DOI:** 10.1186/s13104-018-3807-6

**Published:** 2018-10-04

**Authors:** Nigist Tilahun Teka, Assefa Mulu Baye

**Affiliations:** Department of Pharmacology and Clinical Pharmacy, College of Health Sciences, Addis Ababa University, Addis Ababa, Ethiopia

**Keywords:** Counselling practice, Community pharmacists, Addis Ababa

## Abstract

**Objective:**

Pharmacists monitor diabetic patients more often than any other healthcare providers. It is important that they have appropriate counseling practice on diabetes mellitus management. The aim of this study is to assess the counseling practice of community pharmacists for diabetes mellitus patients in Addis Ababa, Ethiopia.

**Results:**

Among 300 community pharmacy professionals, 58.3% were male. Nearly half of the community pharmacy professionals delivered appropriate counselling service on the appropriate time to administer each oral anti-diabetic drug and missed oral dose. Only 15.3% of the community pharmacy professionals gave proper counselling on the importance of continuous screening for nephropathy, retinopathy, and neuropathy.

## Introduction

Diabetes mellitus (DM) is a metabolic disorders characterized by hyperglycemia and abnormalities in carbohydrate, fat, and protein metabolism. It results from the metabolic defects in insulin secretion, insulin sensitivity, or both [1].

Type 1 DM accounts for 5–10% of all diabetes cases [2]. Type 2 DM accounts for majority of DM cases. It occurs when an unhealthy lifestyle (excessive calories, inadequate exercise, and obesity) is superimposed upon a susceptible genotype [3].

The role of pharmacist has altered drastically over the past three decades. Traditionally pharmacists were viewed as individuals who dispensed medical products to the community. The clinical pharmacy grew with the concept of clinical pharmacy services. The ultimate goal of pharmaceutical care is to optimize and improve a patient’s medical condition and quality of life [4].

Community pharmacists are the healthcare providers most accessible to the public. Their professional activities cover counselling of patients at the time of dispensing of prescription and non-prescription drugs, provision of drug information to health professionals, patients and public, and participation in health-promotion programs including rational use of drugs, alcohol abuse, tobacco use, drug use during pregnancy, poison prevention and treatment and prevention and management of chronic diseases [5].

Community pharmacists are ideally placed to support in the screening, education and referral of individuals at risk of diabetes. Patients with various symptoms contact community pharmacists and, when indicated, pharmacists refer patients to medical practitioners for further management [6]. They are widely accessible, available, in frequent contact with the public and able to access people who are apparently healthy [7].

Researches indicated that most of the community pharmacies professionals lack time, human resources, therapeutic knowledge and confidence that resulted in low level of counseling service [8, 9].

## Main text

### Methods

#### Study setting, design and sampling

The study was conducted at community pharmacies, found in Bole Sub City, Addis Ababa, Ethiopia. The study was conducted from April 2018–June 2018. At the time of the study, there were 122 pharmacies in Bole Sub City. The Sub City has the highest number of community pharmacies and pharmacists in Addis Ababa. An institutional based cross-sectional study design was used.

As the number of pharmacy professionals is limited in the study area, all of the consenting community pharmacists and pharmacy technicians working in the sub city were invited to participate in this survey. The number of community pharmacists practicing in the study area was 310; all the community pharmacists were included in the study. Pharmacy professionals who were the owner of the community pharmacy were excluded to decrease bias associated with misleading and non-genuine information.

#### Study tools

Standardized and structured questionnaire was adopted from relevant literature [9] for the purpose of data collection. The questionnaire was self-administered after consent and clear instruction was communicated verbally.

#### Data analysis

The cleaned and edited data was entered into SPSS version 23 for analysis. Descriptive statistics with percentage of counseling practice and perceived barriers were identified. Data was presented with tables with frequency and percentage.

#### Study procedures

The overall counseling practice was computed from those who counselled always on the following nine diabetes related counseling points [11]. (1) The importance of regular exercise, weight control, and diet. (2) The causes, symptoms and management of hypoglycemia. (3) The appropriate administration, handling, and storage of insulin. (4) The impact of OTC and herbal drugs. (5) Good foot and eye care techniques. (6) Continuous screening for nephropathy, retinopathy, neuropathy. (7) Smoke cessation. (8) The appropriate time to administer each oral anti-diabetic drug and missed oral dose. (9) Matter of stress, tension therefore. All these gave a total score of 9 if a pharmacy professional would counsel on all the above counseling points. Community pharmacy professionals who counselled always on 5 or above of the counseling points were considered with good counseling practice whereas those who counselled always in less than 5 of the above counseling points were considered with poor counseling practice.

### Result

#### Socio-demographic characteristics

Among 310 community pharmacists approached, 300 completed the questionnaire, which gives a response rate of 96%. Among the respondents, 175 (58.3%) were male. The socio-demographic characteristics of the community pharmacists are shown in Table 1.

**Table 1:** Socio-demographic characteristic of the pharmacy professionals in Addis Ababa, 2018

Characteristics	Frequency (%)
Age (years)	
Mean (SD)	34.23 (7.38%)
< 26	27 (9%)
26–36	185 (61.7%)
37–47	71 (23.7%)
> 47	17 (5.7%)
Gender	
Male	175 (58.3%)
Female	125 (41.7%)
Highest pharmacy degree achieved	
Diploma	79 (26.3%)
Bachelor’s degree	204 (68.0%)
Masters	17 (5.7%)
Work experience in community pharmacy (years)	
< 4	88 (29.3%)
4–8	118 (39.3%)
8–12	34 (11.3%)
12–16	44 (14.7%)
< 16	16 (5.3%)
Monthly income (in USD)	
< 127	61 (20.3%)
127–180	80 (26.7%)
181–218	82 (27.3%)
> 218	77 (25.7%)

USD, United States of America Dollar; SD, standard deviation

#### Counseling practice

Counseling on the importance of continuous screening for nephropathy, retinopathy, neuropathy and counseling on good foot care techniques were rarely practiced by 41.3% and 34.3% of community pharmacists, respectively. Three-fourth (76%) of the community pharmacy professionals had ‘poor counseling practice’ for diabetic patients. Counseling practice of community pharmacists for diabetes patients is indicated in Table 2.

**Table 2:** Counseling practice done by community pharmacy professionals for diabetes patients in Addis Ababa, 2018

Counseling topics	Always	Sometimes	Depends on the day	Rarely	Never
Importance of regular exercise, weight control, and diet	81 (27%)	154 (51.3%)	15 (5%)	42 (14%)	8 (2.7%)
Cause, symptoms and treatment of hypoglycemia	47 (15.7%)	130 (43.3%)	20 (6.7%)	87 (29%)	16 (5.3)
Appropriate administration, handling, and storage of insulin	223 (74.3%)	66 (22%)	7 (2.3%)	2 (0.7%)	2 (0.7%)
Impact of OTC and herbal drugs	84 (28%)	141 (47%)	27 (9%)	42 (14%)	6 (2%)
Good foot care, eye care techniques	55 (18.3%)	105 (35%)	31 (10.3%)	103 (34.3%)	6 (2%)
Importance of continuous screening for nephropathy, retinopathy, neuropathy	46 (15.3%)	74 (24.7%)	24 (8%)	124 (41.3%)	32 (10.7%)
Smoke cessation (when applicable)	138 (46%)	104 (34.7%)	24 (8%)	20 (6.7%)	14 (4.7%)
Appropriate time to administer each oral anti-diabetic drug and missed oral dose	146 (48.7%)	104 (34.7%)	26 (8.7%)	24 (8%)	–
Matter of stress, tension, and other illness	57 (19%)	114 (38%)	60 (20%)	67 (22.3%)	2 (0.7%)

#### Barriers of counseling services

‘Lack of time’ was the most frequently perceived barrier (82%) followed by ‘patients are not interested in preventive activities’ with 72%. However, compared to other barriers, ‘lack of knowledge or clinical skills’ was not a barrier to most of the community pharmacy professionals having the lowest percentage of 31.7%. Perceived barriers of counseling services are indicated in Table 3.

**Table 3:** Perceived barriers of counseling for diabetes patients by pharmacy professional in Addis Ababa, 2018

Barriers	Yes	No
Lack of knowledge or clinical skills	95 (31.7%)	205 (68.3%)
Lack of personnel or resources	203 (67.7%)	97 (32.3)
Lack of clinical tools	161 (53.7%)	139 (46.3%)
Lack of coordination with other health care professionals	175 (58.3%)	125 (41.7%)
Lack of access to additional training programs	214 (71.3%)	86 (28.7%)
Lack of time	247 (82.3%)	53 (17.7%)
Patients are not interested in preventive activities	216 (72%)	84 (28%)

### Discussion

Since DM patients visit pharmacies more frequently for different reasons, this creates an opportunity for community pharmacists to counsel and educate the patient regarding the management and counseling on screening, smoke cessation, weight control and regular exercise, healthy diets and prevention of complications. Current and past smoking are associated with a risk of diabetes mellitus in both men and women [1].

In this research, the most frequently counselled diabetic care service was on the appropriate administration, handling, and storage of insulin (74.3%) while counseling on weight control, diet and exercise was only 27% (Table 2).

A research that was done in Katmandu showed that 44% of the community pharmacy professionals always counseled on the importance of exercise and diet control [9]. These findings are also consistent with a study in Scotland [10]. A research done in Ethiopia also explored that the most frequently counseled diabetics care service were on the importance of exercise diet and weight control (32.5%) [11].

The optimal delivery of counseling services for diabetic patients on diet, exercise and weight control are very promising as nutrition therapy and physical activity can improve glycemic control in diabetic patients [12–14]. A significant increase in the proportion of patients whose A1c was less than 7% was observed by the Asheville Project [15].

In the current study, community pharmacists’ counseling practice on the importance of continuous screening for nephropathy, retinopathy, neuropath was found to be relatively lower (15.3%). Similar finding was reported in Qatar [15]. Community pharmacists have the role to encourage diabetic patients’ awareness and screening of these complications.

The most frequently perceived barrier was lack of time. This could be due to high burden of customers or shortage of pharmacy personnel, which is also one of the barriers, that is highly frequent in the community pharmacies. Compared to other barriers, ‘lack of knowledge or clinical skills’ was not a barrier to most of the community pharmacy professionals. The community pharmacists may not able to evaluate their knowledge and clinical skill due to the lower level of the actual counseling practice. Similarly, lack of time was one of the perceived barriers in a study done in Qatar [16]. Increasing the number of pharmacy professionals and establishing private counseling areas in community pharmacies, and promoting the role of pharmacist in diabetes care can help in overcoming these barriers. As indicated by the Asheville project, diabetic patients’ clinical and humanistic outcomes could be improved when they are provided with the pharmaceutical care services of community pharmacists, with no concomitant increase in total direct health care costs [15].

### Conclusion and recommendation

According to this study, community pharmacy professionals had demonstrated a lower level of involvement in counseling services for patients with DM. Low interest of patients for preventive activities and lack of time and personnel are the most perceived barriers to a good counseling practice. Provision of advanced on-job training for community pharmacists and enforcing the deployment of more personnel resource is recommended.

## Limitations

The use of a self-administered questionnaire, which depends on honesty and faith of the respondents, could affect the responses as it may be subjected to social desirability bias although it was not clearly observed by this research. As a descriptive cross- sectional study, it was not possible to identify determinant risk factors for poor counseling practice in community pharmacies.

## Abbreviations

DM: diabetes mellitus
ETB: Ethiopian Birr
IRB: Institutional Review Board
OTC: over the counter
